# Improving the Functionality of Proso Millet Protein and Its Potential as a Functional Food Ingredient by Applying Nitrogen Fertiliser

**DOI:** 10.3390/foods10061332

**Published:** 2021-06-09

**Authors:** Honglu Wang, Dongmei Li, Chenxi Wan, Yan Luo, Qinghua Yang, Xiaoli Gao, Baili Feng

**Affiliations:** 1 State Key Laboratory of Crop Stress Biology in Arid Areas, College of Agronomy, Northwest A&F University, Yangling 712100, China; 2018050099@nwsuaf.edu.cn (H.W.); dmL@nwafu.edu.cn (D.L.); wancx0712@126.com (C.W.); 18220813910@163.com (Y.L.); qinghuayang@nwafu.edu.cn (Q.Y.); gao2123@nwsuaf.edu.cn (X.G.); 2 Shaanxi Research Station of Crop Gene Resources & Germplasm Enhancement, Ministry of Agriculture, Yangling 712100, China

**Keywords:** proso millet, protein, solubility, emulsion, foam

## Abstract

Nitrogen is required for proso millet growth and has a critical influence on yield and quality. However, the effect of nitrogen fertilisation on proso millet protein properties remains unclear. This study aimed to investigate how nitrogen fertiliser treatment (180 kg/hm^2^) affects the structural and functional properties of proso millet protein. In comparison with the control group (N0), nitrogen fertiliser treatment loosened the dense structure of the protein and presented a larger particle size. Nitrogen treatment did not change the main subunit composition, and β-sheet and α-helix were the main secondary structures of proso millet protein based on Fourier transform infrared spectroscopy. In addition, nitrogen fertiliser treatment improved the content of hydrophobic amino acids and β-sheet proportion from proso millet protein, and high water/oil absorption capacity and thermal stability was observed, but the solubility, emulsion stability and foaming properties from proso millet protein decreased. Proso millet proteins exhibited high amino acid content and good functional properties, including solubility, foaming capacity and emulsifying properties, especially the w139 variety. Results show that proso millet protein has great potential for food applications. The above results provide useful information for the food industry to determine emerging gluten-free protein resources.

## 1. Introduction

Global food security issues have increased the focus on sustainable foods and the de-termination of fungible sources of plant protein, making it essential for the overall transi-tion of food and nutrition security [1]. As a substitute for animal-derived protein, plant protein is the main dietary protein for most developing countries [2]. The food industry mostly uses plant proteins isolated from soybeans, rice and wheat, which are economi-cally attractive, sustainable and ethical. Noteworthily, these plant protein resources will not meet the future demand considering the rapid growth of the global population [3]. Therefore, new plant protein resources should be determined. With this trend, research on alternative plant protein resources with proso millet, quinoa and lentils has attracted widespread interest amongst scientists [4].

Proso millet is an important source of emerging plant protein. It can efficiently use water, allowing tolerance to drought and high temperature and growth in barren and high-salinity soil. When the production of other crops is reduced or ceased by natural disasters, proso millet can be grown as a harvest plant, because it has a short growth pe-riod of 10–11 weeks [5]. Most countries consume proso millet, which is the sixth most important grain in the world. From a nutritional perspective, the nutritive parameters of proso millet are comparable or better than those of other cereals. Proso millet protein may prevent liver damage, because it has a beneficial effect on cholesterol metabolism [6]. Pro-so millet has a protein content of 9.5–17%, which is similar or higher to wheat and other cereals, and compared with wheat protein, proso millet protein is rich in essential amino acids, such as isoleucine, methionine and leucine [7]. In addition, proso millet protein is a gluten-free protein, making its application essential in the gluten-free market [8]. For the wide use of proso millet protein in the food market, protein functionality should be thor-oughly understood. Protein is the key ingredient to maintain the food structure through foaming, gelation and emulsification [9], and protein with high gel temperature is an ideal ingredient for meat analogues, where good foaming qualities and high water/oil absorp-tion capacity are ideal for torta, breads and sausages [10]. The functional properties of protein are the basis for product formulation and food processing and are critical for de-termining its potential uses. However, the application of proso millet protein or its protein part as a food ingredient is lacking, possibly because of limited information about its physical and chemical properties, structure and functionality.

Nitrogen is an important and non-negligible nutrient element for crop growth, and appropriate nitrogen content can maintain and improve crop quality [11,12]. The effects of nitrogen fertiliser on starches have been widely studied [13,14], but few studies have de-scribed how nitrogen application affects functional properties of proso millet protein. Therefore, this study aimed to examine the effect nitrogen fertiliser on the physical and chemical properties, structure and functionality of proso millet protein. We analysed the functionality of protein, such as protein solubility, water holding, oil absorption and emulsification capacity, emulsion stability, foaming capacity and foam stability. The amino acid profile, SDS-PAGE, morphology, secondary structure and thermal properties were also evaluated. This research lays a theoretical foundation for the wide application of proso millet protein in the food industry and provides a basic reference for its high-quality cultivation.

## 2. Materials and Methods

### 2.1. Plant Materials and Experimental Design

The proso millet varieties used in the experiments were waxy-139 (w139) variety and non-waxy-297 (n297) variety. Field experiments were conducted at the experimental site in Northwest A&F University (37°56′26″ N, 109°21′46″ E), Yulin city, Shaanxi Province, China in 2020. The soil type was sandy loam, and the 0–20 cm of soil was obtained to measure the soil nutrients following the method of Zhang et al. [15]. The specific soil nutri-ents were as follows: 5.32 g/kg of organic matter, 0.34 g/kg of total N content, 0.59 g/kg of total phosphorus (P), 19.81 g/kg of total potassium (K) and pH 8.69. The experiment was conducted using randomised block design. The N treatment group (N180) was treated with 180 kg/hm^2^ of nitrogen, while the control group (N0) was not treated with nitrogen. All groups were applied with 100 kg/hm^2^ P and 75 kg/hm^2^ K. Nitrogen (urea, N > 46%) was applied in the form of base fertiliser, and no topdressing was applied during proso millet growth. Proso millet was sown on 12 June and harvested on 1 October.

### 2.2. Protein Fractions Analysis

Protein fractions were determined as described by Mao et al. (2014) [16]. Protein was ex-tracted sequentially with distilled water (albumin), 0.5 mol/L NaCl (globulin), 75% etha-nol (prolamin) and finally with 0.1 mol/L NaOH (glutelin) for 30 min at 50 °C. The protein fraction and protein content of samples were determined using the Kjeldhal method with a protein–nitrogen coefficient of 6.25.

### 2.3. Extraction and Isolation of Proso Millet Protein

Proso millet seeds were shelled, defatted, and then ground with universal high-speed smashing machines (FW100, Taisite Ltd., Tianjin, China) and sieved with a 100-mesh sieve to obtain flour. The defatted proso millet flour samples were dispersed in distilled water (1:10, *w*/*v*) and bathed in beaker at 50 °C. After reaching a specified temperature, the pH was adjusted to 8.0 with 0.5 mol/L NaOH and stirred for extraction for 30 min at 50 °C. The mixture was centrifuged at 4000 rpm for 10 min to remove starch deposits. The supernatant was adjusted to pH 3.8 with 0.5 mol/L HCl and centrifuged at 4000 rpm for 10 min. The precipitate was washed thrice with 15 mL of distilled water and 50% ethanol solution and centrifuged at 4000 rpm for 10 min and then collected. The precipitate was freeze-dried in vacuum for 24 h to obtain the protein flour of proso millet. The flour was collected and placed in a refrigerator at 4 °C.

### 2.4. Amino Acid Profile

The protein was hydrolysed at 121 °C with 6 mol/L HCl for 24 h, and 200 μL of protein acid hydrolysate was purged using nitrogen gas. Then 200 μL of 0.1 mol/L HCl extract was added. The solution was mixed well, vortexed and centrifuged. The supernatant was obtained for use. The amino acids were pre-column derivatised using AccQ·Tag Ultra. Finally, the amino acid content was determined using the HPLC system [17].

### 2.5. SDS-PAGE

The main subunits and molecular weight of the protein were determined using the SDS-PAGE method reported by Zha et al. (2019) [18] with slight modification. Polyacrylamide gel electrophoresis was performed with a 10% separating gel and 5% stacking gel. 

### 2.6. Morphological Observation and Granule Size Analysis

The micrograph of protein was obtained using the nona Nano SEM450 scanning electron microscope at an accelerating voltage of 5 kV with 10,000× and 20,000× magnification. The particle size distribution was evaluated using a laser diffraction instrument (Mastersizer 2000, Malvern, UK) [19].

### 2.7. Fourier Transform Infrared Spectroscopy (FTIR)

The secondary structures of proso millet protein were analysed using the FTIR spectrometer (Nicolet iZ-10, Thermo, Weltham, MA, USA). Briefly, 0.05 mg of protein was ground into the power with KBr and then placed into a pellet. The spectral scanning range is 4000–400 cm^−1^, the spectral resolution is 4 cm^−1^ with 32 scans. The content analysis of secondary structure followed the method of Yu, et al. (2021) [20].

### 2.8. Functional Properties of Proteins

#### 2.8.1. Protein Solubility (PS), Water Holding Capacity (WHC) and Oil Absorption Capacity (OAC)

Exactly 0.1 g of protein was placed in tubes and added with 25 mL of NaOH (0.1 mol/L). The solution was homogenised at 15,000 rpm for 2 min, held for 30 min and centrifuged at 4000 rpm for 5 min. The protein concentration of supernatant (*C*_0_) was measured using the Biuret method. The NaOH solution was replaced with a mixed solution (pH 7.5 phosphate buffer, 3 g/100 mL of NaCl and 1 g/100 mL of sucrose), and the above steps were repeated to measure the protein content *C*_1_. The PS was calculated using Equation (1) as follows:

(1) $$\mathrm{PS}\;(\%)=\frac{c_{1}}{c_{0}}\times 100$$

Exactly 0.1 g of protein sample was mixed with 5 mL of mixed liquor in centrifuge tube, thoroughly stirred using glass rod, and then centrifuged at 2500 rpm for 10 min. The supernatant was removed, and the remaining pellet was weighed. The WHC was calculated using Equation (2) as follows:

(2) $$\mathrm{WHC}\;(\%)=\frac{\left(m_{2}-m_{1}\right)}{m_{0}}\times 100$$

where *m*_1_ = weight of tube and sample (g), *m*_2_ = weight of tube and residue after adsorption of water (g) and *m*_0_ = weight of the protein (g).

Exactly 0.2 g of protein was dispersed with 2 mL of salad oil in a centrifuge tube and held for 1 h at room temperature. Then, the mixture was centrifuged at 3500 rpm for 20 min and the supernatant was removed. After 10 min, the centrifuge tube and residue were weighed. The OAC was calculated using Equation (3) as follows:

(3) $$\mathrm{OAC}\;(\%)=\frac{\left(m_{2}-m_{1}\right)}{m_{0}}\times 100$$

where *m*_1_ = weight of tube and sample (g), *m*_2_ = weight of tube and residue after adsorption of oil (g) and *m*_0_ = weight of the protein (g).

#### 2.8.2. Emulsification Capacity (EC) and Emulsion Stability (ES)

Exactly 0.1 g of protein sample was mixed with 15 mL of mixed liquor by using a homogeniser at 12,000 rpm for 2 min and then added with 15 mL of salad oil for homogenisation at 12,000 rpm for 2 min. Exactly 10 mL of emulsion was immediately transferred into a graduated centrifuge tube and centrifuged at 2500 rpm for 5 min. Then, the height of the emulsified layer was determined. The emulsion was heated at 80 °C for 30 min, and the emulsified layer height was determined after cooling. The EC and ES were calculated using Equations (4) and (5) as follows:

(4) $$\mathrm{EC}\;(\%)=\frac{H_{1}}{H_{0}}\times 100$$

(5) $$\mathrm{EC}\;(\%)=\frac{H_{2}}{H_{1}}\times 100$$

where *H*_2_ is the height of the emulsified layer, *H*_1_ is the height of emulsified layer in the tube and *H*_0_ is the height of the total emulsion in the tube.

#### 2.8.3. Foaming Capacity (FC) and Foam Stability (FS)

Exactly 10 mL of mixture (3%, *w*/*v*) was homogenised at 12,000 rpm for 2 min, and the volume was noted before and after stirring. After 3 min, the foam volume was measured. The FC and FS were calculated using Equations (6) and (7) as follows:

(6) $$\mathrm{FC}\;(\%)=\frac{V_{1}}{V_{0}}\times 100$$

(7) $$\mathrm{FS}\;(\%)=\frac{V_{2}}{V_{1}}\times 100$$

where *V*_2_ is volume after 3 min of standing, *V*_1_ is the volume after stirring and *V*_0_ is the volume before stirring.

### 2.9. Thermal Properties

Protein thermal properties were determined via differential scanning calorimetry (DSC; Q 2000, TA Instruments, Wood Dale, IL, USA). Exactly 3 mg of protein flour and 9 μL of water were added into an aluminum pan. The sample pan was heated to 40–150 °C at a rate of 10 °C/min. The parameters recorded were denaturation peak temperature (Td), onset temperature (To), and endset temperature (Te) [20].

### 2.10. Statistical Analysis

All samples were expressed as the mean standard deviation of triplicate measurements. One-way analysis of variance and Duncan test were used for multiple comparisons by using the SPSS 23.0 Statistical Software Program. Statistical significance was considered at *p* ≤ 0.05.

## 3. Results and Discussion

### 3.1. Protein Fractions

**Figure 1:**
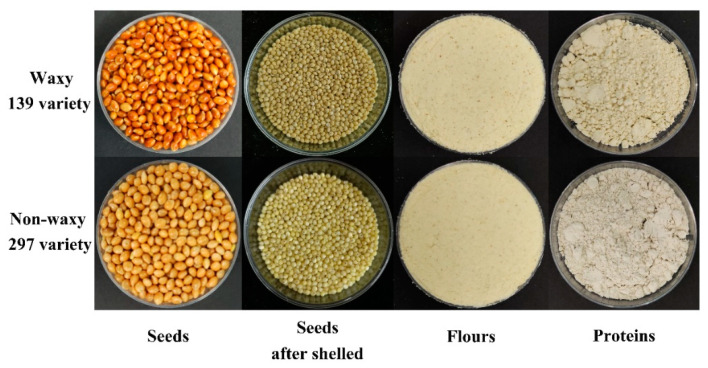
The protein isolated from proso millet seeds.

As shown in Figure 1, proso millet seeds were husked, defatted and then ground to prepare the flour. The flours were used to extract the proso millet proteins. The protein content and protein fractions of proso millet flour and protein content in protein are shown in Table 1. The protein contents of proso millet significantly increased by using nitrogen fertiliser, and with increasing nitrogen levels the protein contents increased from 15.89% to 17.18% (w139) and 15.03% to 18.16% (n297), respectively. Nitrate reductase and glutamine synthetase are the key enzymes for protein synthesis. Increasing nitrogen application can maintain high nitrate reductase and glutamine synthetase activities during grain filling, thus promoting protein synthesis [21]. Based on the extracted fractions, prolamin accounts for 4.05% and 4.36%, while glutelin accounts for 0.29% and 0.48% of w139 variety. For the n297 variety, prolamin accounts for 4.06% and 4.25%, while glutelin accounts for 0.35% and 0.42%. These results (Table 1) agree with other findings for the protein fractions in millet [22,23]. Prolamin and glutenin usually accumulates in the endosperm in the form of protein bodies, while albumin and globulin mainly exist in the embryo and aleurone layer, and they eventually get lost a small amount after milling [24]. Prolamin is the most abundant protein component in proso millet protein. Prolamin was synthesised in a large amount at the late growth stage, and its content is high. Albumin was mainly involved in the metabolic activities of enzymes, and it accumulated rapidly at the beginning of grain filling, and then gradually degraded with the growth period [24]. The contents of Lys, Trp and Met were high in albumin, which was closely related to the quality and rheological properties of bread products. In comparison with the control group (N0), nitrogen application significantly increased the content of protein fractions and remarkably affected the w139 variety, but n297 variety had no significant difference. The protein of proso millet was extracted by alkali-soluble acid precipitation extraction. For the proso millet varieties, the protein isolation obtained from n297 variety showed high protein content than the w139 variety. This finding may be related to the characteristics such as fat content and genotype of the variety. The level of fat content will significantly affect the protein content in the extracted protein [25]. The waxy and non-waxy nature of the variety may also account for the difference in protein content.

### 3.2. Amino Acid Analysis

The amino acid profile of proso millet proteins was further analysed (Table 2), because the balance in amino acid profile is essential for the determination of protein quality. In comparison with the control group (N0), the amino acid content of proso millet increased after nitrogen fertiliser treatment, possibly because of the promotion of protein synthesis (Table 1) and the increase of amino acid content. The application time of nitrogen fertiliser and the application ratio of nitrogen and potassium fertiliser had significant effects on the amino acid content [26]. Glu and Arg were the main components of amino acids in the proso millet proteins, accounting for 2.58–3.42 and 3.18–4.83 g/100 g, respectively. Glu was the first step in the synthesis of amino acids by plants, and could play an important role in the subsequent synthesis of amino acids. Therefore, it was the most important amino acid in amino acid metabolism, and its content was relatively high. Proteins with high Glu content can promote liver glycogen synthesis and regulate the balance of skeletal muscle protein [27]. Based on Table 2, Arg had the highest content in proso millet protein, which was consistent with previous reports that as an important component of synthetic protein, Arg is an intermediate outcome of urea nitrogen cycle [28]. High Arg content was observed in the n297 variety. High Arg content may be conducive for the use of protein for the treatment of cardiovascular diseases. Lys is the first limiting amino acid in most cereals, while Lys of proso millet protein was higher than the minimum requirement set by FAO/WHO (2007). Hence, proso millet protein was a good source of nutrition. Glu and Asp are the primary amino acids of seed storage proteins, and they are collectively called acidic amino acids [29]. There was no significant difference in acidic amino acid content between the N treatment group (N180) and the control group (N0). The hydrophobic amino acid content of n297N0 (17.58 g/100 g) and n297N3 (16.31 g/100 g) was higher than that of w139N0 (14.84 g/100 g) and w139N3 (14.67 g/100 g). Hydrophobic amino acids have a greater impact on the functional properties of proteins. The high content of hydrophobic amino acids provides the dense internal structure of protein form, thus improving its thermal stability [30]. Interestingly, among the amino acid types, the content of hydrophobic amino acid was the highest, followed by the uncharged polar, basic, acidic, and hydrophilic amino acids in proso millet protein. In this study, N180 treatment significantly increased the content of EAA, thereby improved the nutritional value of proso millet. As for the EAA composition of proso millet protein (except His and Trp), which met the adult minimum requirement by FAO/WHO (2007) and could be used as a nutritional supplement.

### 3.3. SDS-PAGE

**Figure 2:**
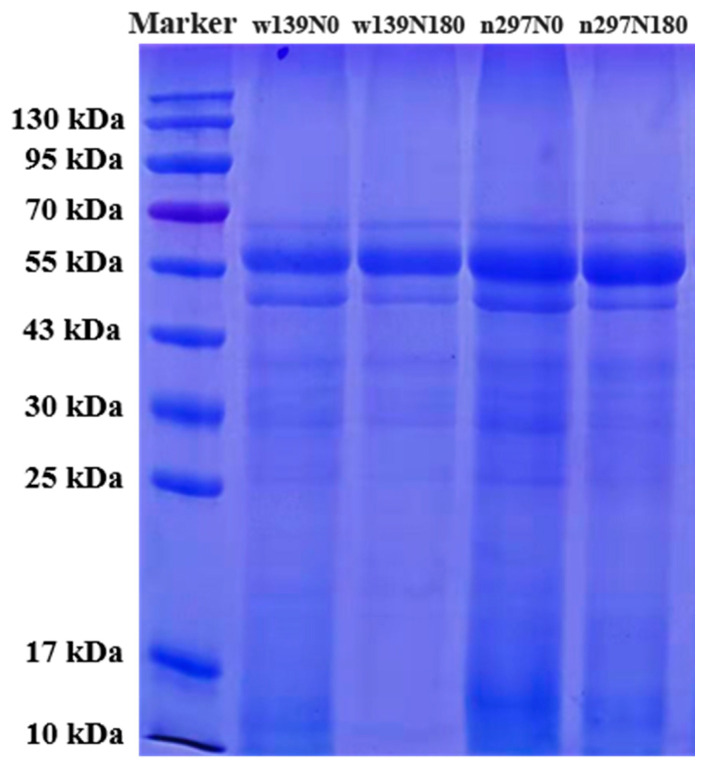
SDS-PAGE protein patterns of proso millet varieties of w139 and n297 under N0 and N180 treatments. The protein mass of each lane is 0.112 mg.

The protein composition of proso millet was evaluated using SDS-PAGE. The protein molecular weights of w139N0, w139N180, n297N0 and n297N180 are shown in Figure 2. The molecular weight of proso millet protein was 10–70 kDa, and it mainly contained six bands. The major subunits observed had molecular weights of 69, 60, 54, 40, 33, 30 and 26 kDa. Most of the subunits observed were significantly consistent from nitrogen fertiliser treatment (N180) and control group (N0). Nitrogen treatment did not change the main subunit composition of proso millet protein. Qiang et al. (2013) [31] reported that freeze-drying and spraying did not change the subunit composition of rice protein. The accumulation of major subunit at 60, 54, 40 and 26 kDa of proso millet protein was dif-ferent, and large accumulation was observed at 60 and 54 kDa in proso millet protein. The two distinct bands at 60 and 54 kDa belonging to the 7S globulin fraction were clearly observed in proso millet protein. The polypeptides with molecular weights of 55 and 65 kDa are 7S globulins [32]. Therefore, proso millet protein is conducive for the recovery of 7S globulins fraction. Proso millet protein exhibited two weak bands at 40 and 26 kDa, corresponding to the 11S globulin fractions of proso millet protein. This result agree with previous findings [33]. Steffolani et al. (2016) [34] observed that molecular weights of ap-proximately 32, 25 and 22 kDa of proteins isolated from some quinoa varieties were varia-tions. The subunits corresponding to molecular weights of 20–22 and 60 kDa correspond to the 11S globulin basic subunit and 7S globulin, respectively [35].

### 3.4. Morphology and Granule Size

**Figure 3:**
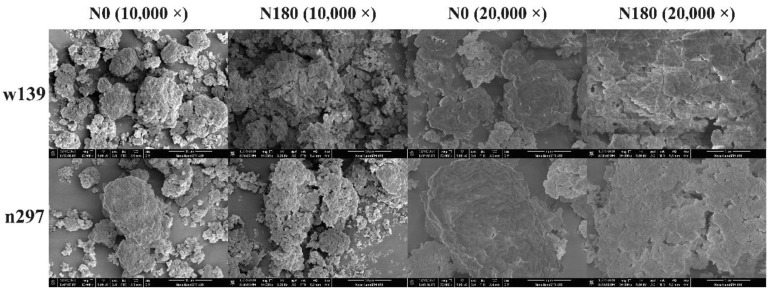
Scanning electron microscope pictures of proso millet varieties of w139 and n297 under N0 and N180 treatments.

The microstructure of proso millet proteins extracted from w139 and n297 varieties are displayed in Figure 3. All protein samples showed irregular rock-like structure and displayed a rough surface with numerous network holes. The freezing of protein caused the formation of ice crystals, which collect the protein molecules together to form a protein mass. Lyophilised protein isolates show irregular protein particles [36]. The observation of protein at 10,000× magnification exhibited that the w139 variety protein particles were smaller and looser than the n297 variety, regardless of the amount of nitrogen fertiliser treatment. In comparison with the control group (N0), nitrogen fertiliser treatment in-creased the small opening on the protein surface and loosened the dense structure of the protein, thus possibly affecting the water holding capacity and protein solubility [37].

**Figure 4:**
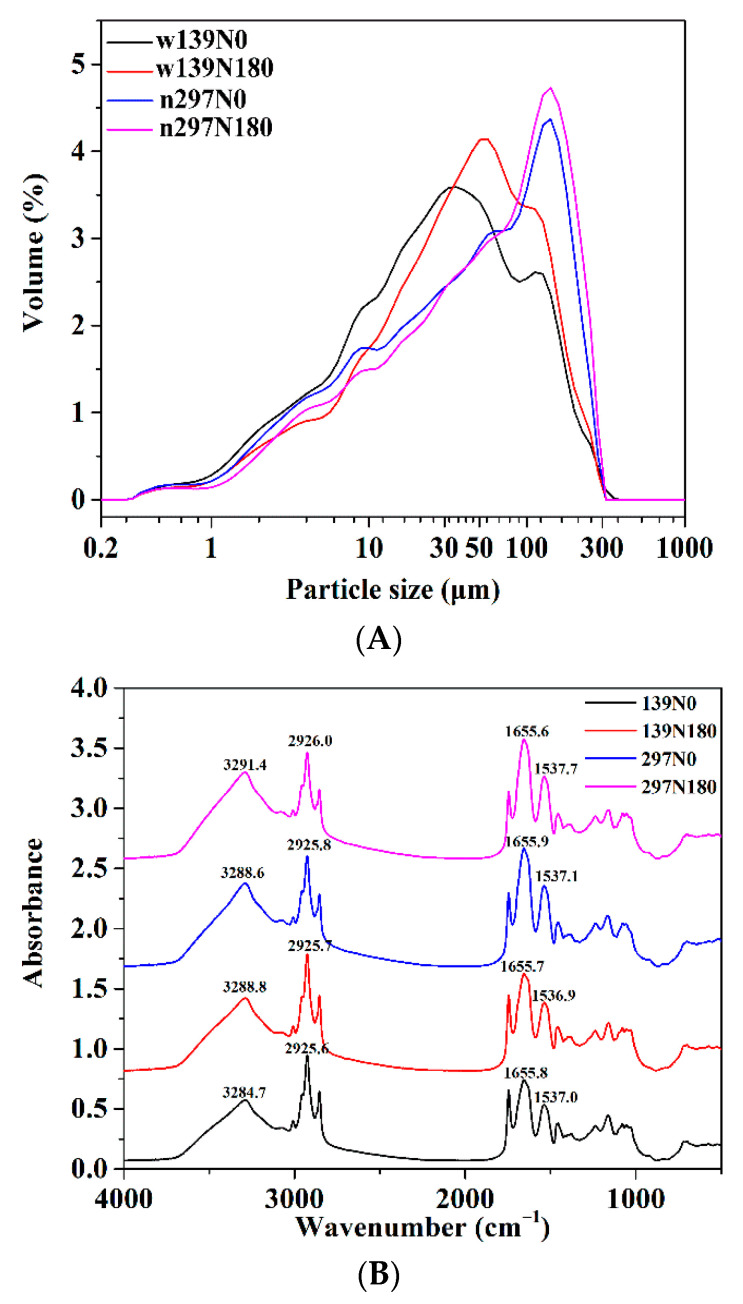
(**A**) The particle size distribution and (**B**) Fourier transform infrared (FTIR) spectra of proso millet varieties of w139 and n297 under N0 and N180 treatments.

Table 3 and Figure 4 show the particle size distributions of the protein samples. All particle size distributions of the protein sample changed markedly after nitrogen fertiliser treatment. All samples treated with nitrogen fertiliser (N180) presented a larger particle size than the control group N0, and the particle size significantly increased. Nitrogen fertiliser application prolonged the grain filling, thus promoting the formation of large protein particles. D [4,3] is the volume occupied by particles, and this parameter is very sensitive to agglomeration and decomposition [38]. The D [4,3] range of the protein is 45.85–73.38 μm and was the highest in n297N180. The n297N0 and n297N180 proteins showed a unimodal distribution of particles, whereas the w139N0 and w139N180 proteins exhibited a bimodal distribution. The particle size and volume distribution of the w139 variety were significantly lower than those of n297 variety, thus supporting the electron microscopy results.

### 3.5. FTIR Spectroscopy

**Figure 5:**
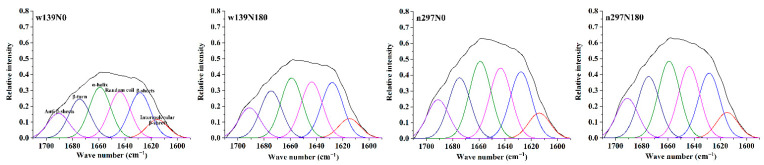
Fourier self-deconvoluted (FSD) curve-fitted spectra of proso millet varieties of w139 and n297 under N0 and N180 treatments.

Figure 4B shows the FTIR spectra of protein in the range of 400–4000 cm^−1^. The curve-fitting spectra of the 1600–1700 cm^−1^ region is shown in Figure 5. Based on Figure 4B, the protein showed wide bands at 3284.7 (w139N0), 3288.8 (w139N180), 3288.6 (n297N0) and 3291.4 cm^−1^ (n297N180) caused by the stretching vibration of O–H from water or protein, while the peaks at approximately 2926 cm^−1^ are assigned to C–H stretching vibration. In all samples, the typical absorption peaks at 1500–1590 (Amide II) and 1600–1710 cm^−1^ (Amide I) correspond to the main peaks of the protein [39]. The peak at 1600–1710 cm^−1^ had the strongest vibration level and is essential to reveal and analyse the secondary structure of proteins. In summary, nitrogen fertiliser treatment will not change the secondary structure of proso millet protein. 

Figure 5 shows that the intermolecular β-sheet, β-sheet, random coils, α-helix, β-turn and antiparallel β-sheet from proso millet protein ranged from 4.71 to 5.56%, 12.35 to 13.61%, 13.38 to 13.76%, 14.40 to 14.90%, 11.23 to 11.72% and 7.12 to 7.71%, respectively. Proso millet protein had the highest proportion of β-sheet and α-helix. More α-helix was observed than β-sheet, thus supporting the findings of Ghumman and Kudre for pulse proteins [40]. This condition might be related to the internal structure of the protein between different crops. Based on Table 3, nitrogen fertiliser (N180) had the highest proportion of β-sheets (13.61%) compared with the control group (N0), indicating that it has a highly ordered structure compared with the control group (N0) [41]. Th abundance of β-sheet conformations result in stable thermal properties of the proteins, resulting in high Td and Δ*H* [10]. In addition, the high proportion of random coil and small amount of β-sheet conformation can improve the protein solubility of the control group (N0). Random structure is easily decomposed by gastrointestinal enzymes to improve gastrointestinal digestibility [41]. Significant differences were observed in all treatments for the β-turns. The β-turn was observed on the surface of protein, and it was in direct contact with the external environment and was the smallest in the secondary structure of protein. However, β-turn can improve the conformational stability of protein through formation in larger secondary structure [42].

### 3.6. Protein Solubility (PS), Water Holding Capacity (WHC) and Oil Absorption Capacity (OAC)

**Figure 6:**
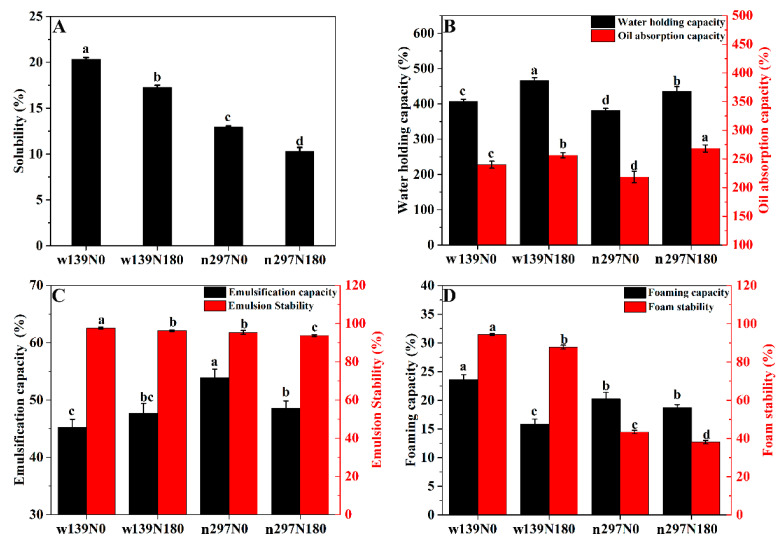
(**A**) Protein solubility, (**B**) water holding capacity and oil absorption capacity, (**C**) emulsification capacity and emulsion stability, (**D**) foaming capacity and foam stability of proso millet varieties of w139 and n297 under N0 and N180 treatments. Different letters (a.b.c.d) represented significant differences.

Solubility is an important property of proteins that affect many functionalities, such as emulsification and foaming. Therefore, protein solubility affects the texture and flavour of food. Figure 6A shows the protein solubility of proso millet, and the solubility was ranged from 9.94 to 20.34%, in which the lowest and highest values were observed in n297N180 and w139N0, respectively. The solubility reduced after nitrogen fertiliser treat-ment compared with the control group (N0, Figure 6A). Generally, the decrease in particle size increases the surface area of the molecule and improves the interaction between pro-tein and water molecules, resulting in high protein solubility [43]. Based on Table 3, all the protein samples of N180 showed a larger particle size than the control group (N0), resulting in low solubility. The solubility of the w139 variety was higher than that of n291 variety, and this property may be attributed to the differences in the amino acid composition and surface hydrophobicity [29]. Thus, the protein of the w139 variety could be suitable as an ideal ingredient for making juice or beverage.

WHC is an important characteristic in determining swelling and water against the gravity of proteins. Figure 6B indicates significant difference in the WHC of proso millet protein. w139N0 and w139N180 had WHC values of 407.21% and 466.21%, while n297N0 and n297N180 had WHC values of 381.41% and 435.09%, respectively. The WHC under nitrogen fertiliser (N180) was higher than that of the control group (N0), thus explaining the higher ratio of polar or hydrophilic amino acids of protein under nitrogen fertiliser (N180) [44], as demonstrated in Table 2. This property depends on the protein structure, amino acid profile and content of hydrophobic amino acids [3]. The WHC of proso millet protein is comparable with those of soybean (1.3 g/g) and pea (1.7 g/g) protein isolates [45]. This difference could be attributed to the grain characteristics, changes in protein conformation and different isolation methods [41]. In the food industry, a WHC content of protein ranging from 1.49 g/g to 4.72 g/g is the best choice for viscous food. Therefore, proso millet protein with high WHC content can become an important component of food industry.

The OAC can measure the ability of protein to interact with lipid. The OAC could be affected by oil applied and hydrophobic amino acid content. In the present study, the OAC of values w139N0, w139N180, n297N0 and n297N180 were 239.92%, 256.52%, 218.64% and 268.42%, respectively. The OAC followed the same trend as WHC. Nitrogen fertiliser treatment (N180) significantly improved the OAC of proso millet protein, thus supporting the SEM results (Figure 3). The SEM shows that nitrogen fertiliser treatment (N180) is light and porous, which was beneficial to the absorption of fat, while the control group (N0) had a dense protein structure, which hindered fat from entering the protein matrix. Deng et al. (2019) [29] reported that the prominent OAC is positively correlated with its high hydrophobicity. The OAC of proso millet protein is similar to that of quinoa protein (2.76 g/g) [46] but is higher than that of pea (1.20 g/g) and faba bean protein (1.60 g/g) [45]. The high WHC and OAC of proso millet proteins make it an ideal ingredient for different products. In these products, shortening and hydration are important parameters for cake, bread and muffin.

### 3.7. Emulsification Capacity (EC) and Emulsion Stability (ES)

EC represents the ability of protein to form emulsion, while ES measures the stability of protein forming emulsions in a specific time [47]. ES depends on the composition, size, charge and hydrophobicity of protein, while EC depends on the magnitude of their interactions [48]. The EC and ES of proso millet protein are illustrated in Figure 6C. The EC values of w139N0, w139N180, n297N0 and n297N180 were 45.27%, 47.70%, 53.91% and 48.60%, respectively, which significantly differed. The ES of w139N0 was the highest (97.59%) and that of w139N180 was higher (96.27%) than those of n297N0 (95.35%) and n297N180 (93.73%), and the difference was significantly different. The nitrogen fertiliser treatment (N180) proteins of the w139 variety had higher EC than the control group (N0) because of the high content of hydrophobic amino acids, but the control group (N0) had higher ES than the nitrogen fertiliser treatment (N180). Considering the interaction of a large number of hydrophobic amino acids, the binding ability of oil droplet and emulsifier increased, thus improving the emulsifying performance of protein. The proso millet protein had good emulsification properties, making it an excellent emulsifier in baking products. The emulsification properties of proteins, such as EC and ES, are essential for food applications, especially in chopped meat, frozen desserts and mayonnaise. These food products are exposed to different levels of pressure during preparation. Thus, different emulsifying and stabilising capacities are required.

### 3.8. Foaming Capacity (FC) and Foam Stability (FS)

Proteins are regarded as good foaming agents. The foaming properties of FC and FS are important for mixing and aeration in the food industry [49]. The effects of nitrogen fertiliser on the FC and FS of proso millet protein are shown in Figure 6D. The FC values of the w139 and n297 varieties were in the range of 15.80–23.62% and 18.68–20.22%, respectively, while those of FS were 87.83–94.41% and 38.11–43.38%, respectively. The FC and FS of control group (N0) were significantly higher than those of nitrogen fertiliser treatment (N180), supporting the highest solubility (Figure 6A) and smaller particle size (Table 3) in the control group (N0). Excellent FC depends on the high level of protein solubility. Li et al. (2020) [28] have reported that higher protein solubility can enhance the interaction between water and protein and help in protein structure stretching, thus providing good seal and contributing to foam formation. Proteins with small particle size such as the w139N0 protein can be absorbed quickly during stirring and form more foam [31]. In the present study, the FS of w139 variety was significantly higher than that of n297 variety. A good FS forms a viscous, elastic, viscous, airtight and continuous film around air bubbles. The w139 variety is more suitable in the food industry such as in whipped toppings, in which stable whipping properties are needed.

### 3.9. Thermal Properties

DSC was used to study the thermal functional properties of proso millet protein under different nitrogen levels. Table 4 shows the To, Td and Te values. Table 4 shows that the Td of proso millet proteins ranged from 106.99 °C to 111.08 °C, which was significantly higher than that of other plant proteins such as mung bean (~86.23 °C) [50] pea (~85 °C) [51] and flaxseed protein (~85 °C) [52]. Hence, proso millet proteins could be added as good additives to the heat-processed food system to maintain its function. Significant difference in N0 was observed between the two varieties of proso millet. However, nitrogen application significantly affected the thermal properties of proso millet protein, and the thermal stability of nitrogen fertiliser treatment (N180) was significantly better than that of control group (N0). The perfect thermal stability of nitrogen fertiliser treatment (N180) could be attributed to the interaction between hydrophobic amino acids, thus supporting that nitrogen fertiliser treatment (N180) contained a high proportion of hydrophobic amino acids [29]. The high Td of the nitrogen treatment is attributed to the strong hydrophobic interaction between the protein and protein molecule, causing the protein to form a dense microstructure and extended β-sheets, which can be proved by the secondary structure of the protein [53]. The presence of lipids could also hinder the hydrophobic interactions between protein molecules and enhance the hydrophobic binding between lipids and protein molecules, thus reducing the thermal stability. A similar phenomenon that thermal properties depend on lipid content was also observed in buckwheat protein [54].

## 4. Conclusions

In comparison with the control group (N0), nitrogen application significantly increased the content of protein fractions, but the w139 variety was more sensitive to nitrogen than the n297 variety. The EAA composition of proso millet protein (except His and Trp) met the minimum requirement for adults. Thus, it can be used as a nutritional supplement. Nitrogen fertiliser treatment loosened the dense structure of the protein and presented a large particle size. Nitrogen treatment did not change the main subunit composition of proso millet protein, and the β-sheet and α-helix were the main secondary structures of proso millet protein based on FTIR. In addition, nitrogen fertiliser treatment improved the content of hydrophobic amino acids and β-sheet proportion from proso millet protein, resulting in high water holding capacity, oil absorption capacity and thermal stability. The high content of hydrophobic amino acids provided the dense internal structure of the protein, thus improving its thermal stability. The protein from the w139 variety had higher protein solubility, water holding capacity, foaming capacity and foam stability than the n297 variety. The application of nitrogen fertiliser affected the amino acid profile and secondary structure of proso millet protein, thus affecting its functionality such as protein solubility, emulsification capacity and foaming capacity. Finally, the protein quality of proso millet variety changed.

## Figures and Tables

**Table 1:** Effect of nitrogen fertilizer on protein content and protein fractions of proso millet flour and protein content in isolated proso millet protein ^a^.

Varieties	Flour					Isolated Proso Millet Protein
	Protein (%)	Albumin (%)	Prolamin (%)	Glutelin (%)	Globulin (%)	Protein (%)
w139N0	15.89 ± 0.07c	1.45 ± 0.05b	4.05 ± 0.02b	0.29 ± 0.01c	2.32 ± 0.08c	61.80 ± 0.17c
w139N180	17.18 ± 0.22b	1.65 ± 0.05a	4.36 ± 0.05a	0.48 ± 0.01a	2.67 ± 0.02a	64.05 ± 1.35c
n297N0	15.03 ± 0.42d	1.39 ± 0.07b	4.06 ± 0.03b	0.35 ± 0.05ab	2.44 ± 0.07b	73.27 ± 1.65b
n297N180	18.16 ± 0.27a	1.60 ± 0.02a	4.25 ± 0.23ab	0.42 ± 0.03a	2.43 ± 0.03b	76.57 ± 1.24a

^a^ Data are means ± standard deviations, *n* = 3. Values in the same column with different letters are significantly different (*p* < 0.05).

**Table 2:** Effect of nitrogen fertilizer on amino acid profile and essential amino acids score (%) of proso millet protein.

Amino Acids	Proso Millet Protein Isolate (g/100 g Protein)				FAO/WHO ^a^
	w139N0	w139N180	n297N0	n297N180	Adult
Asp	2.14 ± 0.16a	2.22 ± 0.20a	2.31 ± 0.03a	2.32 ± 0.07a	
Ser	1.63 ± 0.06c	1.81 ± 0.08b	1.89 ± 0.03b	2.03 ± 0.04a	
Glu	2.58 ± 0.15a	2.98 ± 0.23b	3.42 ± 0.05a	3.40 ± 0.06a	
Gly	2.17 ± 0.06d	2.40 ± 0.06c	2.52 ± 0.03b	2.70 ± 0.01a	
Ala	1.95 ± 0.09b	2.15 ± 0.12a	2.15 ± 0.02a	2.27 ± 0.05a	
Gys	0.24 ± 0.01c	0.33 ± 0.01b	0.34 ± 0.01b	0.44 ± 0.01a	
Tyr	1.31 ± 0.05d	1.41 ± 0.04c	1.57 ± 0.01b	1.74 ± 0.02a	
Arg	3.18 ± 0.09d	3.83 ± 0.06c	4.47 ± 0.03b	4.83 ± 0.02a	
Pro	1.98 ± 0.08c	2.22 ± 0.10b	2.30 ± 0.02b	2.50 ± 0.04a	
Trp	-	0.01 ± 0.00	-	-	0.50
Thr	1.24 ± 0.06c	1.38 ± 0.07b	1.34 ± 0.02b	1.48 ± 0.03a	0.90
Val	1.99 ± 0.09c	2.13 ± 0.09b	2.17 ± 0.04b	2.33 ± 0.05a	1.30
Met	0.65 ± 0.02c	0.73 ± 0.02b	0.73 ± 0.02b	0.84 ± 0.01a	1.70
Ile	1.27 ± 0.05b	1.30 ± 0.06b	1.29 ± 0.02b	1.42 ± 0.06a	1.30
Leu	2.60 ± 0.09c	2.84 ± 0.14b	2.78 ± 0.06bc	3.03 ± 0.09a	1.90
Phe	2.06 ± 0.07c	2.11 ± 0.04c	2.37 ± 0.04b	2.49 ± 0.03a	1.90
His	1.22 ± 0.06d	1.39 ± 0.05c	1.47 ± 0.03b	1.63 ± 0.01a	1.60
Lys	1.90 ± 0.12a	2.13 ± 0.26a	1.98 ± 0.04a	2.08 ± 0.05a	1.60
ARM ^b^	3.36d	3.52c	3.94b	4.23a	1.90
Hydrophobic ^c^	14.67c	15.87b	16.31b	17.58a	
Hydrophilic ^d^	4.42c	4.93b	5.14b	5.69a	
Basic ^e^	6.30d	7.52c	7.92b	8.54a	
Acidic ^f^	4.72b	5.2ab	5.73a	5.72a	
Up ^g^	6.59c	7.33b	7.66b	8.39a	
EAA ^h^	12.93c	14.02b	14.13b	15.30a	

^a^ Essential amino acids: requirement by FAO/WHO (2007) for adult. ^b^ Aromatic amino acids, Phe and Tyr. ^c^ Hydrophobic amino acids: Ala, Val, Ile, Leu, Phe, Pro, Gly and Met. ^d^ Hydrophilic amino acids: Ser, Thr, Cys, Tyr. ^e^ Basic amino acids: Arg, His and Lys. ^f^ Acidic amino acids: Asp and Glu. ^g^ Uncharged polar: Gly, Cys, Thr, Tyr and Ser. ^h^ Essential amino acids: Trp, Thr, Val, Met, Ile, Leu, Phe, His, and Lys. Different letters (a.b.c.d) represented significant differences.

**Table 3:** Effect of nitrogen fertilizer on granule size distribution of proso millet protein ^a^.

Varieties	d (0.1) ^b^	d (0.5)	d (0.9)	D [3,2]	D [4,3]
w139N0	3.52 ± 0.06d	27.02 ± 0.42d	116.65 ± 1.84d	8.34 ± 0.09d	45.85 ± 0.45d
w139N180	4.58 ± 0.09b	36.79 ± 0.31c	126.99 ± 0.32c	10.39 ± 0.11b	53.39 ± 0.49c
n297N0	3.91 ± 0.06c	43.44 ± 0.39b	160.42 ± 0.80b	9.44 ± 0.15c	65.16 ± 0.27b
n297N180	4.88 ± 0.06a	52.97 ± 0.16a	174.77 ± 0.33a	11.18 ± 0.25a	73.38 ± 0.24a

^a^ Data are means ± standard deviations, *n* = 3. Values in the same column with different letters are significantly different (*p* < 0.05). ^b^ The d (0.1), d (0.5) and d (0.9) are the granule size at which 10%, 50% and 90%of all the granules by volume are smaller. The D [3,2] and D [4,3] are the surface-weighted and volume-weighted mean diameter, respective.

**Table 4:** Effect of nitrogen fertilizer on proportion of secondary structures and thermal properties of proso millet protein ^a^.

Varieties	Secondary Structure Composition						Thermal Properties		
	Intermolecular β-Sheets (%)	β-Sheets (%)	Random Coils (%)	α-Helices (%)	β-Turns (%)	Antiparallel β-Sheets (%)	To (°C)	Td (°C)	Te (°C)
w139N0	5.56 ± 0.18a	13.04 ± 0.09b	13.76 ± 0.07a	14.73 ± 0.02b	11.23 ± 0.08d	7.12 ± 0.11c	100.36 ± 0.48b	106.99 ± 0.43d	138.57 ± 0.67c
w139N180	4.71 ± 0.02c	13.61 ± 0.08a	13.38 ± 0.03c	14.61 ± 0.02c	11.49 ± 0.02c	7.39 ± 0.02b	101.86 ± 0.21a	110.04 ± 0.43bc	141.87 ± 0.15b
n297N0	4.95 ± 0.05b	12.35 ± 0.20c	13.52 ± 0.04b	14.90 ± 0.05a	11.61 ± 0.07b	7.51 ± 0.03ab	99.81 ± 0.10b	107.89 ± 1.42cd	141.69 ± 2.03b
n297N180	4.79 ± 0.02bc	12.94 ± 0.06b	13.43 ± 0.02c	14.40 ± 0.03d	11.72 ± 0.04a	7.71 ± 0.23a	102.27 ± 0.82a	111.08 ± 2.54a	147.32 ± 0.25a

^a^ Data are means ± standard deviations, *n* = 3. Values in the same column with different letters are significantly different (*p* < 0.05).

## Data Availability

Data are contained within the article.

## Abbreviations

w139	Waxy-139
n297	Non-waxy-297
SEM	Scanning electron microscope
FTIR	Fourier transform infrared spectroscopy
PS	Protein solubility
WHC	Water holding capacity
OAC	Oil absorption capacity
EC	Emulsification capacity
ES	Emulsion stability
FC	Foaming capacity
FS	Foam stability
DSC	Differential scanning calorimetry
Td	Denaturation peak temperature
To	Onset temperature
Te	Endset temperature
